# Surgical Strategy Based on Radiological 3D Reconstruction in a Giant Metastatic Neuroendocrine Tumor of the Pancreas: A Case Report of an Interdisciplinary Approach

**DOI:** 10.1155/2021/8811155

**Published:** 2021-01-25

**Authors:** Gabriel Fridolin Hess, Savas Deniz Soysal, Guillaume Nicolas, Martin Bolli, Christoph Johannes Zech, Alexandar Tzankov, Emanuel Christ, Michael Montemurro, Otto Kollmar

**Affiliations:** ^1^Clarunis University Center for Gastrointestinal and Liver Disease, St. Clara Hospital and University Hospital Basel, Spitalstrasse 21, CH-4056 Basel, Switzerland; ^2^Department of Radiology and Nuclear Medicine, University Hospital Basel, Petersgraben 4, CH-4051 Basel, Switzerland; ^3^Institute of Pathology, University Hospital Basel, Schönbeinstrasse 40, CH-4056 Basel, Switzerland; ^4^Institute of Endocrinology, Diabetology & Metabolism, University Hospital Basel, Petersgraben 4, CH-4051 Basel, Switzerland; ^5^Department of Oncology, University of Lausanne, Rue du Bugnon 46, CH-1011 Lausanne, Switzerland

## Abstract

**Background:**

Neuroendocrine tumors (NETs) are a rare entity and are most commonly found in the gastroenteropancreatic tract. The clinical outcome depends on the potential resectability, grade, and stage. Here, we report a case of a tumor debulking in a metastatic NET of the pancreas. A 25-year-old woman with stable metastatic NET of the pancreas G2 T4N1M1 (hepatic, extrahepatic) already underwent several therapies. *Case Presentation*. A 25-year-old woman with stable metastatic NET of the pancreas G2 T4N1M1 (hepatic, extrahepatic) already underwent several pharmaceutical therapies. Due to the young age, the G2 characteristic, and the stable liver disease, the decision for debulking was made. Based on a 3D CT scan, an embolization was successfully performed directly prior to a pylorus-preserving pancreatic head resection, advanced interaortocaval lymph node dissection, and an atypical liver resection within segment VI. Histological workup revealed a stage pT3, G2, pN1 (29/34), pM1c (hepatic and extrahepatic), L1, V0, Pn0 with complete surgical resection of the primary tumor (180 mm). The excision of the liver segment V showed a completely resected metastasis.

**Conclusions:**

In this patient, extensive surgery of a pancreatic NET with the aim of a prolonged progression-free survival was performed. Close cooperation between different disciplines is absolutely mandatory. Modern imaging allowed a precise therapy plan to be worked out.

## 1. Introduction

Neuroendocrine tumors (NETs) are rare tumors with a relative incidence of 2% of all neoplasms and are most commonly found in the gastroenteropancreatic tract [1]. The absolute incidence of these rare neoplasms has increased in recent years. This is mainly due to the improved diagnostics and increasing knowledge about these tumors [1, 2]. The clinical outcome of patients with NETs depends on several factors, such as location and resectability of the tumor, Ki67 expression, which determines grading, and finally staging [3–5]. In case of a neuroendocrine carcinoma, the chemo regimen of choice is usually based on platin salts and etoposide. In well-differentiated high-grade NET, there are multiple approved options, but peptide receptor radionuclide therapy (PRRT) or a chemotherapy with capecitabine and temozolomide (CAP-TEM) has been proven the most effective ones with higher objective response rate than somatostatin analogs. PRRT has also been studied in neoadjuvant setting of pancreas NET [6].

Future disease progression can be predicted by the following factors: proliferative index Ki67, extension of liver metastases, and presence of distant extra-abdominal lesions [3].

## 2. Case Presentation

**Figure 1:**
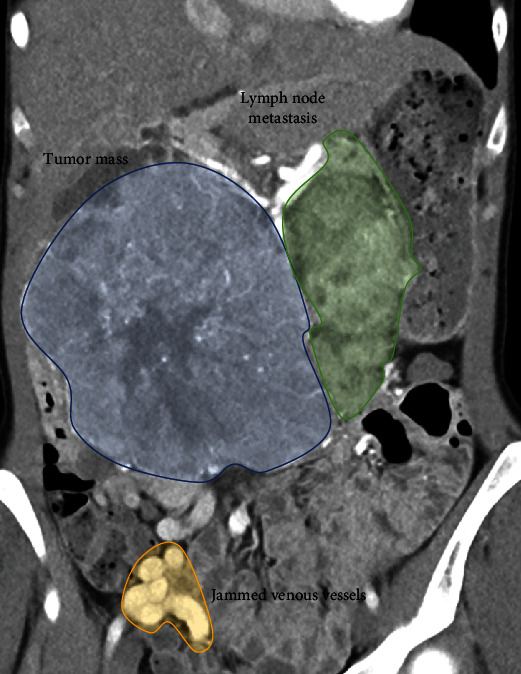
Tumor spread.

**Figure 2:**
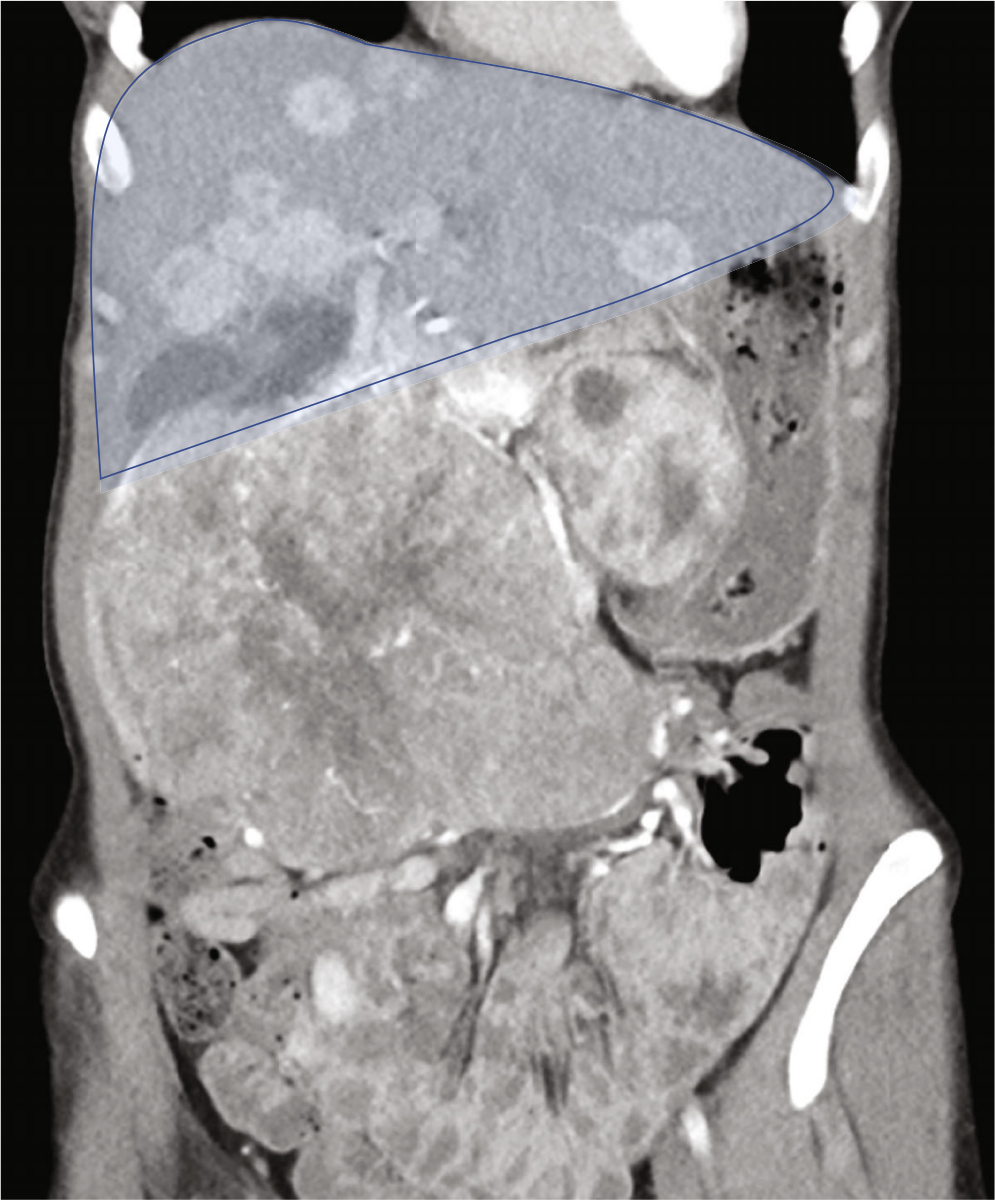
Metastatic liver.

In March 2014, computed tomography (CT) imaging and liver biopsy of a 25-year-old female patient revealed an intermediate grade (G2) NET of the pancreas involving the duodenum [7]. The tumor formation in the right abdomen was manually very well palpable and displaceable with respect to the right kidney. Imaging showed multiple bilobar liver metastases, interaortocaval and para-aortic lymph node metastases, and extra-abdominal thoracic and cervical lymph node metastases (Figures 1 and 2).

At that time, in view of the extensive metastatic spread, let alone the expected extent of surgery for the primary tumor, both the patient and the medical team rejected surgery and in the following years the patient received chemotherapy, peptide receptor radionuclide therapy (PRRT), and since August 2017 lanreotide injections.

In December 2018, the hepatic metastases showed reduced volume by 21%, but the primary tumor had increased by 18% on its largest axis, and furthermore, the patient started to report intermittent symptoms of gastric obstruction. The extra-abdominal metastases were stable. Hepatic biopsy at that time revealed that the metastases were G1, potentially explaining the differential response to the primary tumor, which was G2. Restarting chemotherapy with capecitabine/temozolomide was proposed, but the patient was hesitant and asked for a second and third opinion. A second university hospital and tertiary referral center for NET considered the finding to be inoperable because of a suspected invasion of the mesenteric vessel axis and also proposed capecitabine/temozolomide.

**Figure 3:**
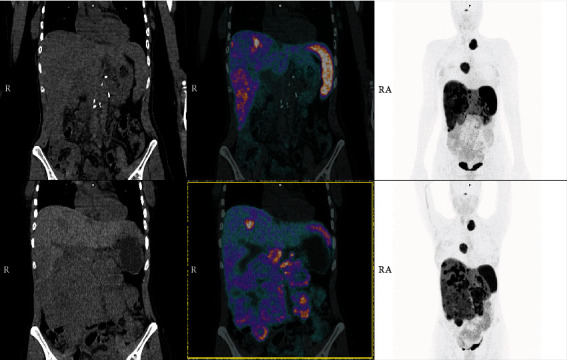
^68^Ga-DOTATATE PET/CT.

In March 2019, the patient was seen in our institution. Based on a repeated somatostatin receptor positron emission tomography-computed tomography (PET-CT) scans, the extrahepatic primary and lymphonodal tumor formation was progressive while the liver metastases remained stable under the therapies mentioned above (Figure 3). Due to the young age, the G2 characteristic, the stable liver disease, and a Ki67 proliferation rate of 5%, our interdisciplinary tumor board advocated for surgical treatment followed by continuous chemotherapy treatment of the liver and extra-abdominal metastases. The aim of the surgical procedure was the debulking of the huge pancreatic tumor mass as well as lymph node dissection. The liver metastases and extrahepatic, thoracic, and cervical lesions were planned to be addressed in a second step.

**Figure 4:**
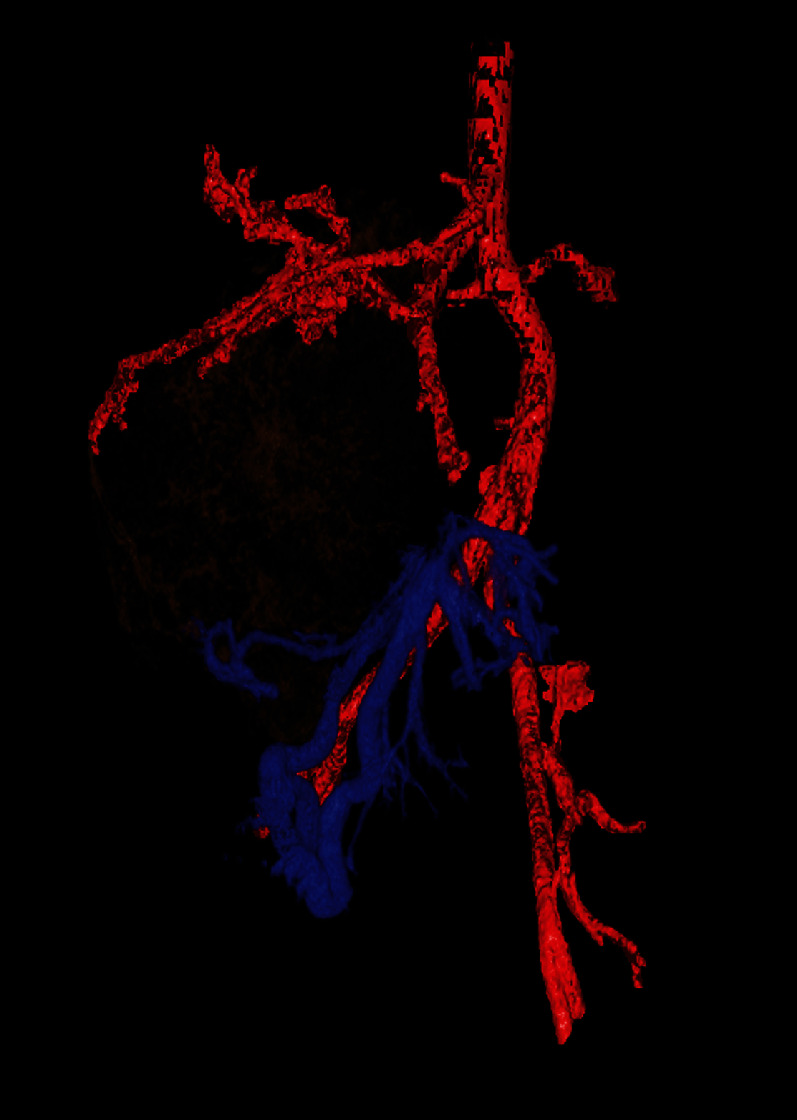
3D reconstruction of the arterial and venous blood supply especially of the tumor mass (brown).

**Figure 5:**
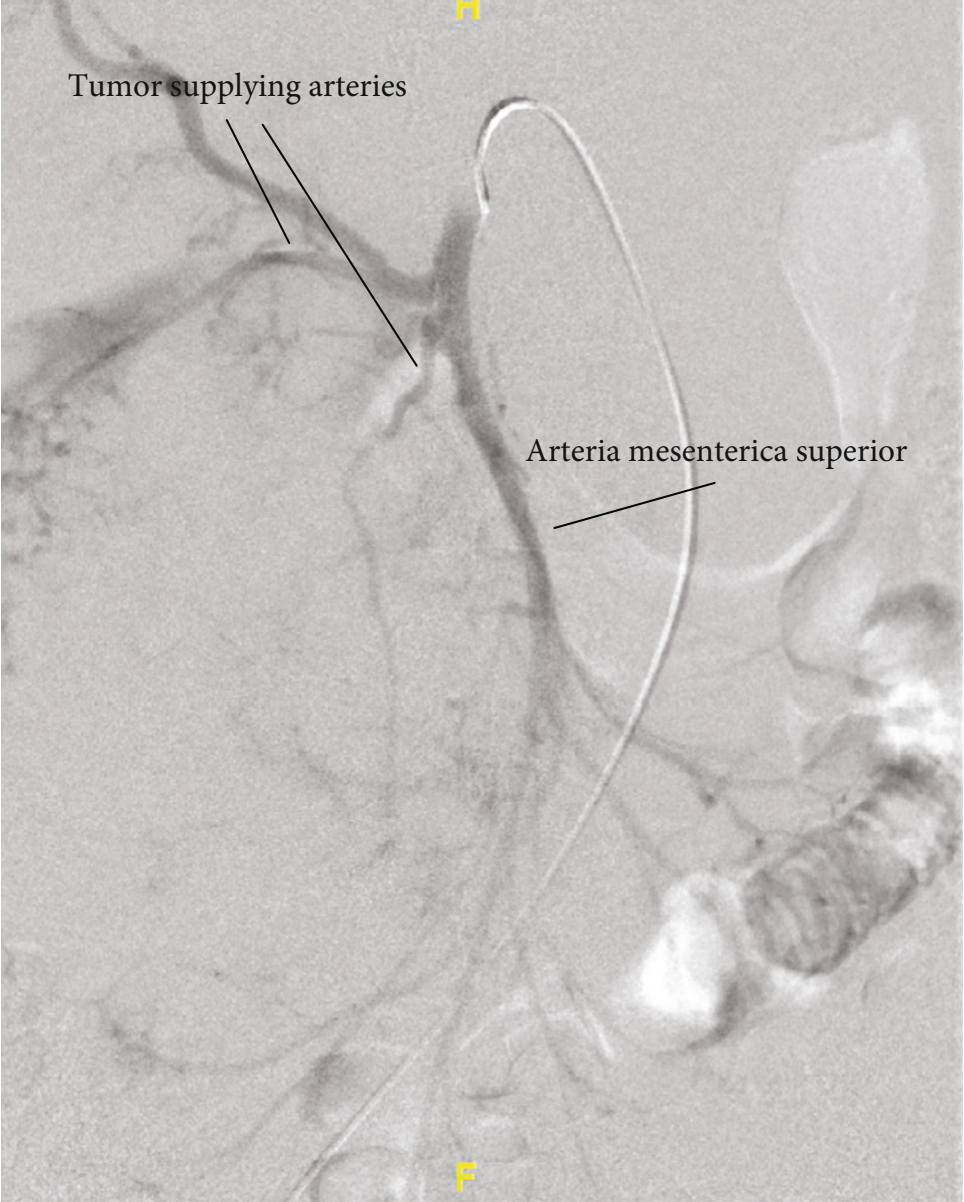
Angiography via a 4F-C2-catheter placed in the arteria mesenterica superior (AMS). Please note the multiple small tumor-feeding irregular and tortuous branches that arise from the AMS. The normal branches for arterial supply of the bowel present as regular, straight vessels (arrow).

As part of the operation planning, a CT scan in order to three-dimensionally present the vascular supply of the main tumor formation was performed (Figure 4). Based on these pictures, a catheter angiography was performed for detailed assessment of the individual tumor branches. A clear arterial supply of the tumor via multiple small- and intermediate-sized arterial feeders arising from the superior mesenteric artery (SMA) could be demonstrated (Figure 5). Therefore, an embolization of the main arterial branches of the tumor with pushable microcoils (Tornado, Cook Medical, Bloomington, US) via a 2.8 F microcatheter (ProGreat, Terumo Europe, Leuven, Belgium) with subsequent resection of the tumor formation was intended.

**Figure 6:**
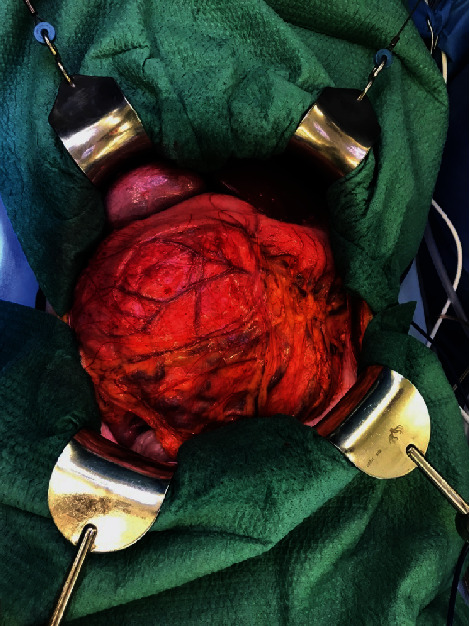
Tumor mass.

**Figure 7:**
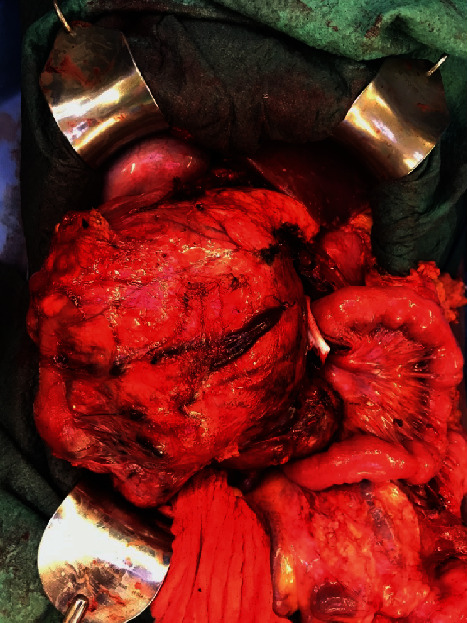
Tumor mass.

**Figure 8:**
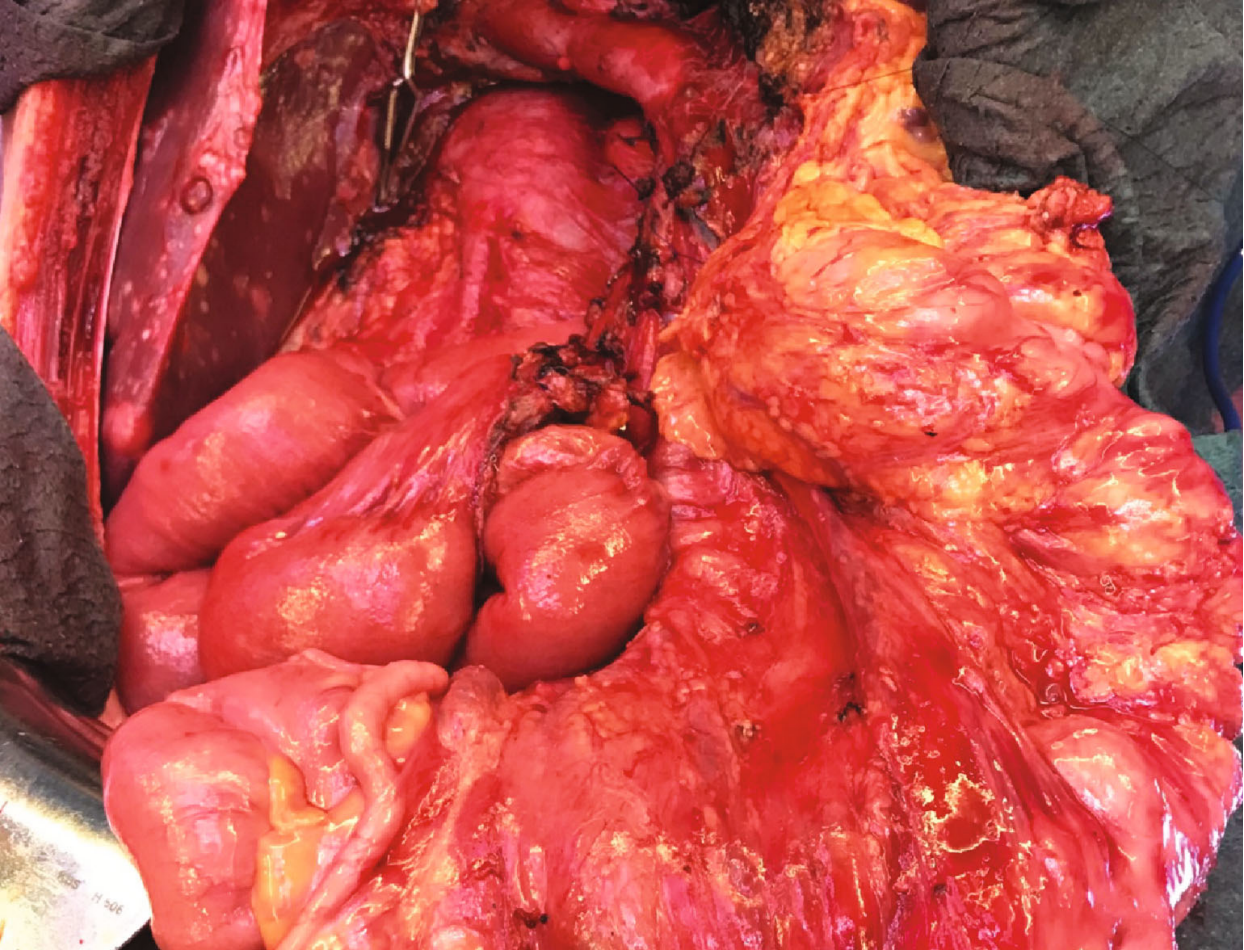
Situs after resection.

**Figure 9:**
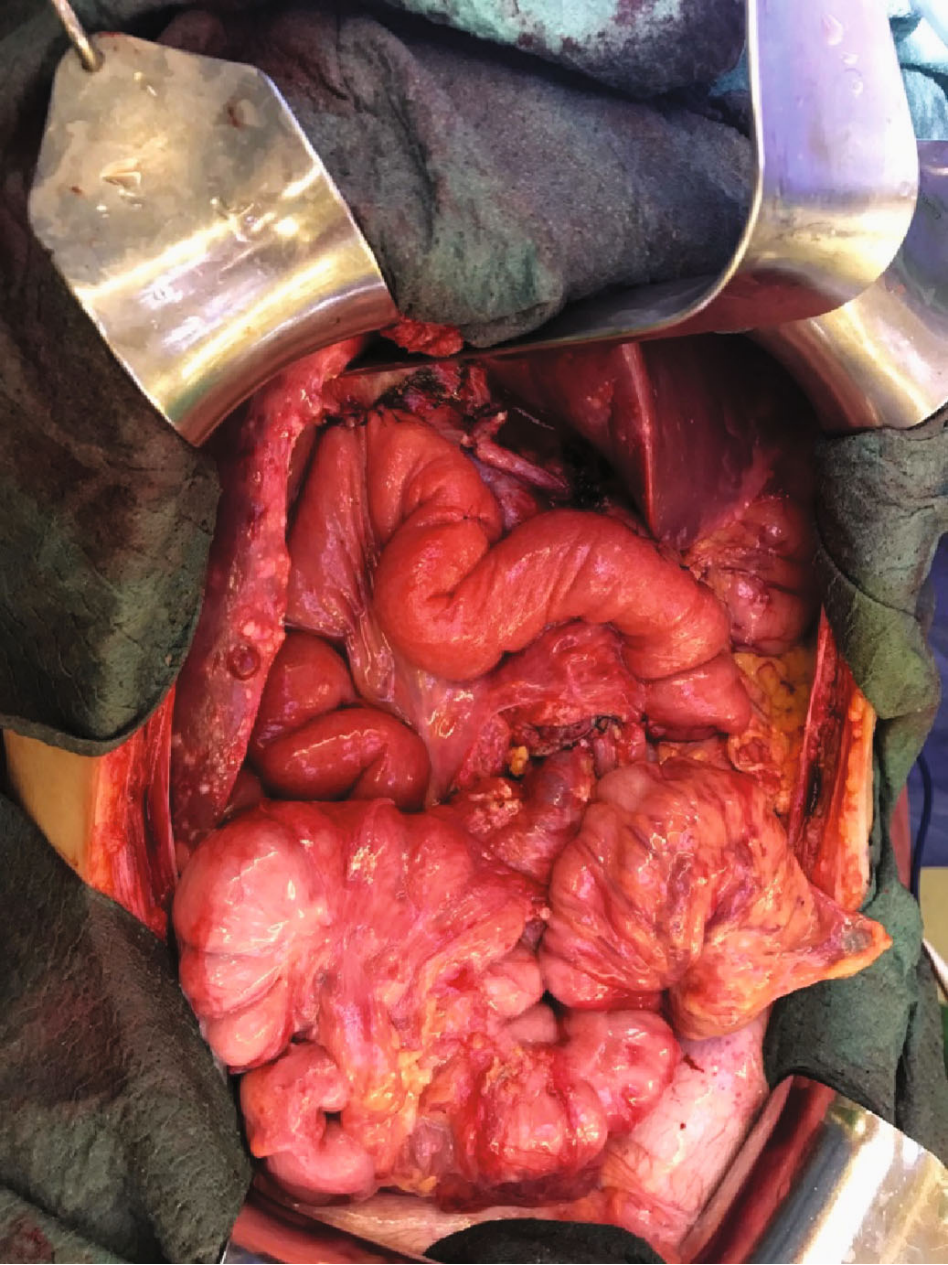
Situs after reconstruction.

The embolization was successfully performed directly prior to a pylorus-preserving pancreatic head resection, advanced interaortocaval lymph node dissection, and an atypical liver resection within segment VI (Figures 6–9). Postoperatively, the patient recovered well and was discharged on the 21^st^ postoperative day.

Histological workup revealed a moderately differentiated neuroendocrine tumor of the pancreas with a maximum tumor diameter of 180 mm. The mean Ki67 proliferation rate of the primary tumor was 9%, whereas the lymph node metastasis showed 15%. TNM classification according to the 8^th^ edition 2017 was pT3, G2, pN1 (29/34), cM1c (hepatic and extrahepatic), L1, V0, Pn0 with complete surgical resection of the primary tumor [8]. The excision of the liver segment VI showed, as expected, a completely resected metastasis.

## 3. Discussion and Conclusions

NETs are rare tumors with a relative incidence of 2% of all neoplasms and are most commonly found in the gastroenteropancreatic tract [1]. The absolute incidence of these rare neoplasms has increased in recent years. This is mainly due to the improved diagnostics and increasing knowledge about these tumors [1, 2]. The clinical outcome of patients with NETs depends on several factors, such as location and resectability of the tumor, Ki67 expression, which determines grading, and finally staging [3–5]. In metastatic endocrine carcinomas of the pancreas, the combination of capecitabine/temozolomide responds well with a positive effect on survival and toxicity compared to streptozocin-based therapies [9].

Future disease progression can be predicted by the following factors: proliferative index Ki67, extension of liver metastases, and presence of distant extra-abdominal lesions [3]. In our patient, Ki67 proliferation after histological workout in the resected specimen was up to three times higher (15%) than in the biopsy that was taken a year before (5%). The difference in the Ki67 mitotic proliferation rate can be explained by the different grading of the main tumor (G2) and metastases (G1) [10]. Our patient had a slow-growing process for five years after the first diagnosis. The decision for surgical resection was due to the context of impending gastric obstruction, the intermittent symptoms of pain and nausea, and the risk of mechanical compression or invasion of major abdominal vessels. The young age, excellent performance, and nutritional status allowed to proceed with surgery to prevent tumor-associated complications such as occlusive ileus, hemorrhage, or organ-specific insufficiency. What we have obtained with surgery is a removal of the primary tumor and a preserved gastric passage.

At the interdisciplinary tumor board, it was decided to control the patient for disease progression by means of a gallium-68-DOTATATE PET/CT six months after surgery. Treatment with somatostatin receptor analog (lanreotide) was continued until two weeks before gallium-68-DOTATATE PET/CT, which showed a stable course, in particular of the two known cervical and thoracic (right hilum) lymph node metastases. A robotic resection of the cervical and thoracic lymph nodes is planned. The liver metastases are as well currently stable under lanreotide therapy and might remain stable for a long time period due to the low grade. In the case of progression, nowadays, there are numerous options to treat even multiple liver metastases. The handling should be performed multimodal, for example, systemic treatment, by selective internal radiation therapy (SIRT), radiofrequency ablation, bland embolization, chemotherapy, or a combination of several techniques.

Panzuto et al. described in their work the risk score for tumor progression. With 15% Ki67, 25–50% liver involvement, and extra-abdominal metastases, our patient would be placed in the high-risk group with a median progression-free survival (PFS) of 12 months [3]. In the literature, there is a tendency for surgery, even in metastatic NETs of the pancreas, always depending on individual patient-based factors [11], especially in young patients who have a longer survival due to their age [12, 13]. Prospective data are currently missing.

Close cooperation between different disciplines and open discussion beyond hospital borders are absolutely mandatory. Modern imaging including somatostatin receptor PET/CT and CT angiography with 3D reconstructions allows to create precise therapy plans. The large tumor debulking of the primary tumor allows therefore, in subsequent steps, to remove the cervical and thoracic metastases. This individual approach does not obtain a situation of absence of disease but limits the spread to the liver with all therapeutic options mentioned above, including liver transplantation in the future as a possibility in the absence of extrahepatic metastases.

## References

[1] Shen H., Yu Z., Zhao J., Li X. Z., Pan W. S. (2016). Early diagnosis and treatment of gastrointestinal neuroendocrine tumors. *Oncology Letters*.

[2] Patel N., Benipal B. (2019). Incidence of neuroendocrine tumors in the United States from 2001-2015: a United States Cancer Statistics analysis of 50 states. *Cureus*.

[3] Panzuto F., Merola E., Pavel M. E. (2017). Stage IV gastro-entero-pancreatic neuroendocrine neoplasms: a risk score to predict clinical outcome. *The Oncologist*.

[4] Panzuto F., Nasoni S., Falconi M. (2005). Prognostic factors and survival in endocrine tumor patients: comparison between gastrointestinal and pancreatic localization. *Endocrine-Related Cancer*.

[5] Panzuto F., Boninsegna L. F. N., Fazio N. (2011). Metastatic and locally advanced pancreatic endocrine carcinomas: analysis of factors associated with disease progression. *Journal of Clinical Oncology*.

[6] Partelli S., Bertani E., Bartolomei M. (2018). Peptide receptor radionuclide therapy as neoadjuvant therapy for resectable or potentially resectable pancreatic neuroendocrine neoplasms. *Surgery*.

[7] Scoazec J.-Y., Couvelard A. (2017). Classification of pancreatic neuroendocrine tumours: changes made in the 2017 WHO classification of tumours of endocrine organs and perspectives for the future. *Annales de Pathologie*.

[8] You Y., Jang J. Y., Kim S. C. (2019). Validation of the 8th AJCC cancer staging system for pancreas neuroendocrine tumors using Korean nationwide surgery database. *Cancer Research and Treatment*.

[9] Strosberg J. R., Fine R. L., Choi J. (2011). First-line chemotherapy with capecitabine and temozolomide in patients with metastatic pancreatic endocrine carcinomas. *Cancer*.

[10] Klimstra D. S., Modlin I. R., Coppola D., Lloyd R. V., Suster S. (2010). The pathologic classification of neuroendocrine tumors: a review of nomenclature, grading, and staging systems. *Pancreas*.

[11] Keutgen X. M., Nilubol N., Glanville J. (2016). Resection of primary tumor site is associated with prolonged survival in metastatic nonfunctioning pancreatic neuroendocrine tumors. *Surgery*.

[12] Franko J., Feng W., Yip L., Genovese E. M. A., Moser A. J. (2010). Non-functional neuroendocrine carcinoma of the pancreas: incidence, tumor biology, and outcomes in 2,158 patients. *Journal of Gastrointestinal Surgery*.

[13] Ito H., Abramson M., Ito K. (2010). Surgery and staging of pancreatic neuroendocrine tumors: a 14-year experience. *Journal of Gastrointestinal Surgery*.

